# Digital single-operator cholangioscopy-guided endoluminal radiofrequency of an intraductal papillary mucinous neoplasia of the main pancreatic duct

**DOI:** 10.1055/a-2161-3286

**Published:** 2023-09-27

**Authors:** Xin Deng, Jingwen Wang, Tong Mou, Long Pan, Bin Li, Chengyou Du, Qiao Wu

**Affiliations:** 1 Department of Hepatobiliary Surgery, The First Affiliated Hospital of Chongqing Medical University, Chongqing, China; 2 Department of Neurology, The First Affiliated Hospital of Chongqing Medical University, Chongqing, China

A 64-year-old woman with a history of recurrent acute pancreatitis was referred for treatment of intraductal papillary mucinous neoplasia (IPMN) that was found during a computed tomography (CT) scan in a local hospital. Magnetic resonance cholangiography (MRCP) presented dilation of the main pancreatic duct and suspicious nodules in the main pancreatic duct ( Fig. 1 ). Endoscopic retrograde cholangiopancreatography (ERCP) showed that the pancreatic duct was significantly dilatated and there seemed to be a cystic lesion at the head of the pancreas ( Fig. 2 ). Further evaluation of digital single-operator cholangioscopy (DSOC) found that the pancreatic duct was full of thick, mucinous fluid, and a few of papillary neoplasms were located at the junction of the main branch and branches of the pancreatic duct in the head of the pancreas ( Video 1 ). Tissues were obtained by biopsy forceps and histopathologically examined.

**Fig. 1:**
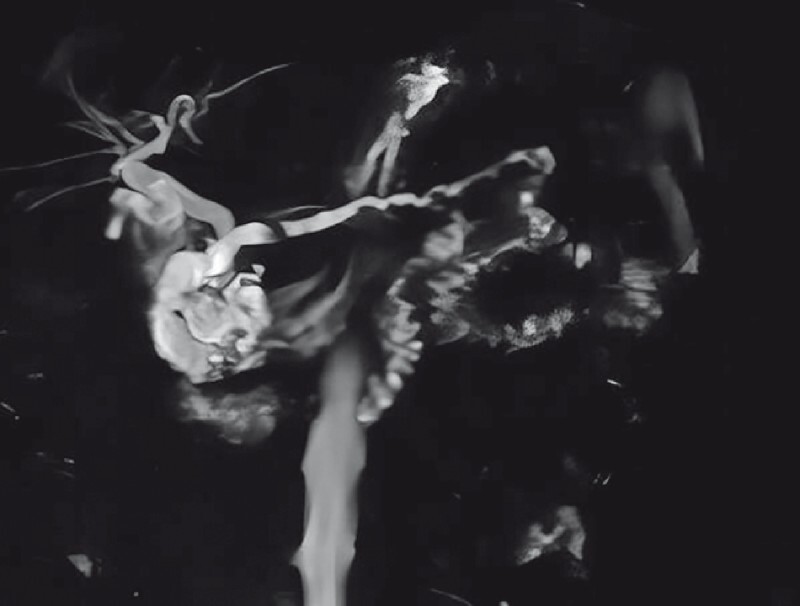
Magnetic resonance cholangiography (MRCP) showed dilation of the main pancreatic duct and suspicious nodules in the main pancreatic duct.

**Fig. 2:**
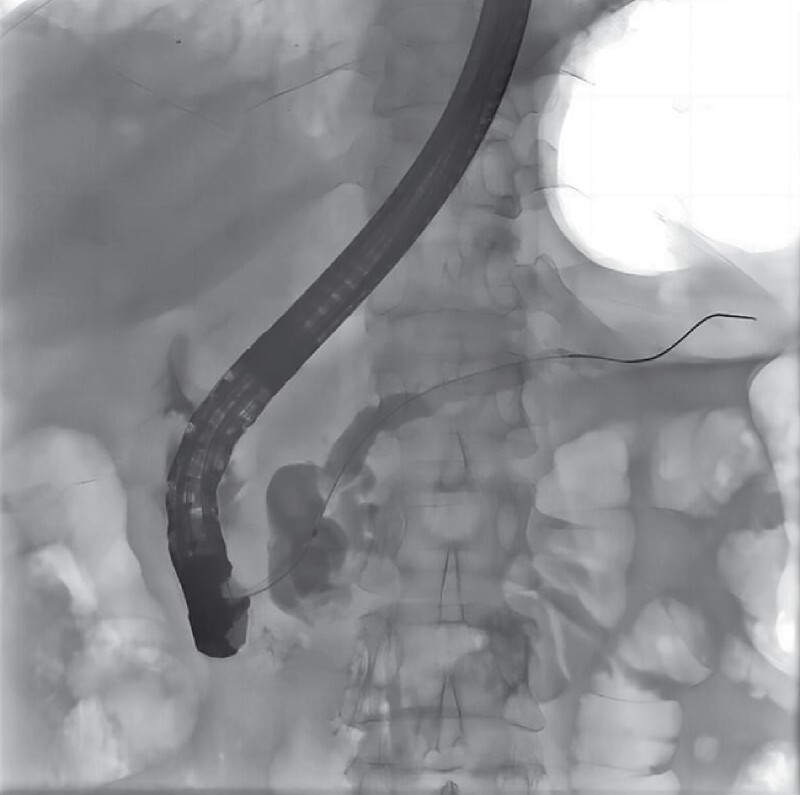
Endoscopic retrograde cholangiopancreatography (ERCP) showed dilation of the main pancreatic duct, which was the same as in the MRCP; however, there were no obvious filling defects.

**Video 1** Digital single-operator cholangioscopy (DSOC) was used to examine the pancreatic duct. Neoplastic tissues were biopsied and then destroyed by endoluminal radiofrequency under direct vision.


The patient and her relatives all refused to have her undergo radical surgery, and intraductal radiofrequency ablation (RFA) was offered. A novel radiofrequency operation electrode was inserted into the pancreatic duct over DSOC ( Fig. 3 ). DSOC-guided endoluminal radiofrequency was used to destroy the neoplasms under direct vision. After radiofrequency ablation, these neoplasms became necrotic ( Fig. 4 ). A 5-Fr stent was placed to prevent secondary stenosis ( Fig. 5 ). No adverse events occurred. Finally, pathology examination revealed IPMN with mild dysplasia. To date, the patient has not developed acute pancreatitis again after 3 months of follow-up.

**Fig. 3:**
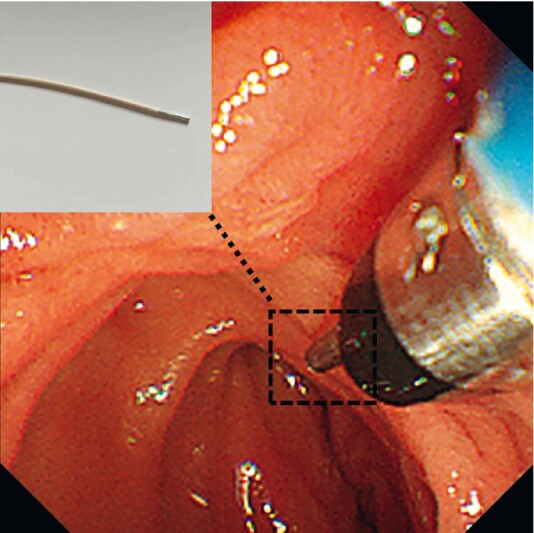
A digital single-operator cholangioscopy (DSOC)-guided endoluminal radiofrequency catheter was inserted into the pancreatic duct. The working channel diameter of this catheter was 1 mm; the working channel length was 5 mm.

**Fig. 4:**
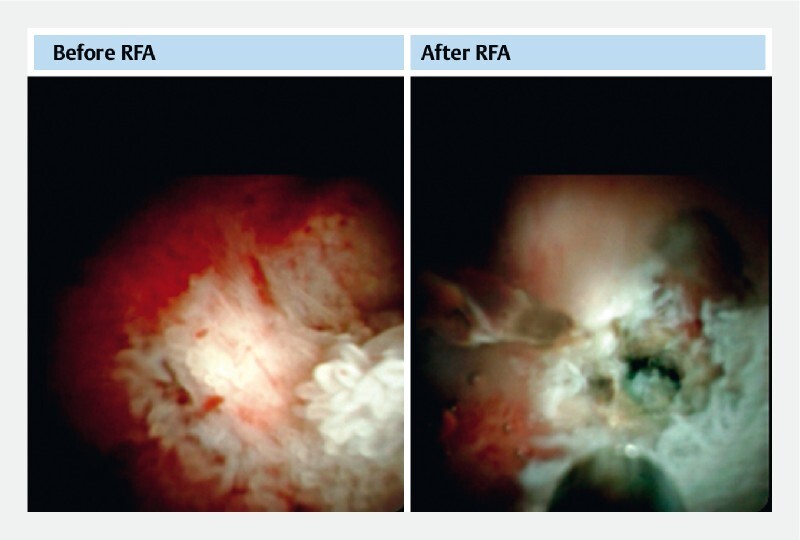
The papillary neoplasms were found by DSOC. After radiofrequency ablation, these neoplasms became necrotic.

**Fig. 5:**
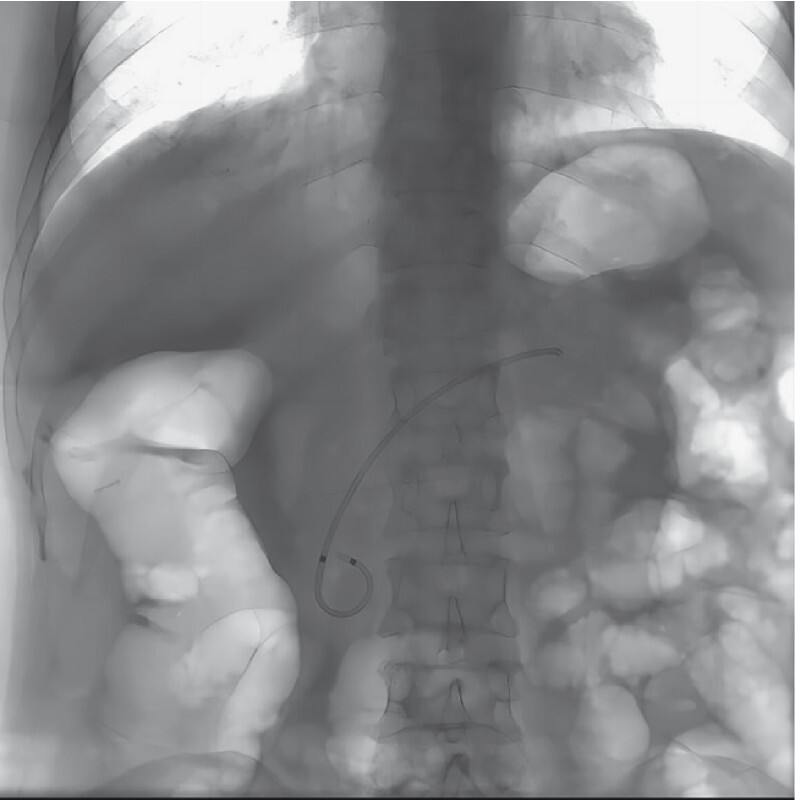
A 5-Fr stent was placed to prevent secondary stenosis and post-ERCP pancreatitis.

RFA ablates neoplastic tissue via local thermal coagulative necrosis 1 . Previous studies have shown that endoscopic biliopancreatic RFA is a safe and effective therapy 2 3 4 . However, these methods are guided by fluoroscopic images. The present case report is the first to report the use of a DSOC-guided endoluminal radiofrequency catheter. The effective outcome and uneventful recovery suggest that this technique could be offered with a curative intent in selected patients. Meanwhile, it could offer a novel, accurate, and microinvasive treatment method for pancreatic duct-related disorders.

Endoscopy_UCTN_Code_TTT_1AR_2AF

## Notes

Endoscopy E-Videos

https://eref.thieme.de/e-videos
*E-Videos* is an open access online section of the journal *Endoscopy* , reporting on interesting cases and new techniques in gastroenterological endoscopy. All papers include a high-quality video and are published with a Creative Commons CC-BY license. Endoscopy E-Videos qualify for HINARI discounts and waivers and eligibility is automatically checked during the submission process. We grant 100% waivers to articles whose corresponding authors are based in Group A countries and 50% waivers to those who are based in Group B countries as classified by Research4Life (see: https://www.research4life.org/access/eligibility/ ). This section has its own submission website at https://mc.manuscriptcentral.com/e-videos

## References

[1] Rustagi T Chhoda A Endoscopic radiofrequency ablation of the pancreas Dig Dis Sci 2017 62 843 850 28160105 10.1007/s10620-017-4452-y

[2] Lorenzo D Barret M Bordacahar B Intraductal radiofrequency ablation of an intraductal papillary mucinous neoplasia of the main pancreatic duct Endoscopy 2018 50 176 177 29169194 10.1055/s-0043-121459

[3] Xia M X Wang S P Yuan J G Effect of endoscopic radiofrequency ablation on the survival of patients with inoperable malignant biliary strictures: A large cohort study J Hepatobiliary Pancreat Sci 2022 29 693 702 33829657 10.1002/jhbp.960

[4] Zheng X Bo Z Y Wan W Endoscopic radiofrequency ablation may be preferable in the management of malignant biliary obstruction: A systematic review and meta-analysis J Dig Dis 2016 17 716 724 27768835 10.1111/1751-2980.12429

